# Memantine prodrug as a new agent for Alzheimer’s Disease

**DOI:** 10.1038/s41598-019-40925-8

**Published:** 2019-03-15

**Authors:** Simona Sestito, Simona Daniele, Deborah Pietrobono, Valentina Citi, Lorenza Bellusci, Grazia Chiellini, Vincenzo Calderone, Claudia Martini, Simona Rapposelli

**Affiliations:** 1 Department of Pharmacy, University of Pisa, Pisa, 56126 Italy; 2 Department of Pathology, University of Pisa, Pisa, 56126 Italy; 3 Interdepartmental Research Centre for Biology and Pathology of Aging, University of Pisa, Pisa, Italy

## Abstract

Hydrogen sulphide has recently drawn much attention due to its potent anti-inflammatory and neuroprotective roles in brain functions. The purpose of the current study was to exploit these beneficial properties of H_2_S to design a new agent for the treatment of Alzheimer’s disease (AD). To pursue our aims, we replaced the free amine group of memantine with an isothiocyanate functionality as a putative H_2_S-donor moiety. The new chemical entity, named memit, was then tested *in vitro* to determine whether it retains the pharmacological profile of the “native drug”, while also providing a source of H_2_S in the CNS. Indeed, Memit showed the ability to release H_2_S through a cysteine-mediated mechanism, thus generating memantine. Moreover, the new hybrid molecule exerts protective effects against neuronal inflammation and induces a drastic fall in ROS production. In addition, memit was also able to reduce the Aβ(1-42) self-induced aggregation and exerted cytoprotective effect against Aβ oligomers-induced damage in both human neurons and rat microglia cells. Finally, similarly to memantine, the new compound promotes autophagy, a complex process required for cellular homeostasis in cell survival that results to be altered in neurodegenerative diseases. In conclusion, our study revealed that memit is a prodrug of memantine. Further *in vivo* studies will be necessary to fully investigate the synergic or cumulative effects due to the H_2_S-releasing moiety and the native drug.

## Introduction

Alzheimer’s disease (AD) is a progressive age-dependent neurodegenerative brain disorder that slowly destroys memory and thinking skills. It is the cause of 60% to 70% of cases of dementia and represent one of the main public health issue with a significant impact on the whole society.

Over the past two decades, it has become increasingly apparent that AD neuropathology is characterized by a dramatic reduction of H_2_S production in the CNS^1^. H_2_S is a gaseous transmitter that functions as a smooth muscle relaxant and neuromodulator. It is produced endogenously from the amino acids L-cysteine and homocysteine (HCy) by several enzymes such as cystathionine β-synthase (CBS), cystathionine γ-lyase (CSE), and 3-mercaptopyruvate sulfurtransferase (3MST) along with cysteine aminotransferase (CAT).

CBS, highly expressed in the hippocampus and cerebellum, is the main enzyme responsible for the production of H_2_S in the brain^2^. Following a neuronal excitation, CBS produces H_2_S which enhances the NMDA receptor-mediated responses thus affecting memory and facilitating the induction of long-term potentiation (LTP)^3^. Recently, a dramatic decrease of CBS activity and consequently a drastic fall in H_2_S levels (about 50%) have been detected in the brain of patients affected by AD, thus suggesting that a reduced production of H_2_S may be involved in the cognitive decline associated to AD^4^.

Nowadays, H_2_S is also considered a neuromodulator^2,5^ since it interacts with N-methyl-D-aspartate (NMDA), a class of ionotropic glutamate receptors which play critical roles in synaptic plasticity. Even though the mechanism is not yet completely understood, H_2_S seems to interact with NMDA receptor both directly, through the sulfhydration of cysteine residues^6^, and indirectly, by regulating the level of intracellular Ca^2+ ^^7^.

Several *in vitro* and *in vivo* experiments have shown the neuroprotective role of H_2_S in AD^8–11^. Growing evidences indicate that NaHS, an exogenous H_2_S-generating agent, induces neuroprotection against oxidative stress (OS) through at least three main mechanisms: (a) restoration of GSH (glutathione) cellular levels^12^; (b) activation of ATP sensitive potassium channels (K_ATP_); (c) reduction of mitochondrial ROS production^13,14^. Recently, it has been also demonstrated that H_2_S has protective effects against Aβ-induced cell injury by inhibiting inflammation, promoting cell growth, and preserving mitochondrial function^2,15^.

In addition, H_2_S exhibits anti-inflammatory and antiapoptotic activities. Indeed, administration of NaHS in a rat model of AD proved to dramatically reduce the release of proinflammatory cytokines such as tumor necrosis factor-α (TNF-α), interleukin IL-1β and IL-6 induced by amyloid β-peptides. Moreover, it inhibits the upregulation of COX2 enzyme and the activation of the nuclear factor-κB (NF-kβ) in the hippocampus^16^, thus reiterating the high potential value of H_2_S in AD therapy. Further H_2_S neuroprotective mechanisms have been suggested: such as the reduction of lipopolysaccharide (LPS)-induced NO production in microglia via inhibition of p38-MAPK pathway^17^.

Taken together all these findings corroborate the functional involvement of H_2_S in neurodegenerative diseases^18^. Therefore, the restoration of the correct levels of endogenous H_2_S is an appealing novel approach for the development of new pharmacological therapies for AD, that are still lacking of an effective treatment. In this paper we decide to investigate the possibility to exploit the H_2_S properties to design a new agent for the treatment of AD, following the multitarget directed ligand strategy (MTDL). This approach is currently used for the design of new molecules to be used in several multifactorial pathologies such as cancer^19^, cardiovascular^20^ and neurodegenerative diseases, including AD^21,22^.

Even though the combination of two or more pharmacophores into a single molecule has been extensively studied, the introduction of an H_2_S-donor moiety into the molecular scaffold of a “native” neuroprotective drug has not been explored yet^9,23–25^. Consistently, to pursue our aim we selected memantine as “native drug” to combine appropriately with a H_2_S-generating moiety. Memantine is an excellent example of neuroprotective drug. It is a well-known uncompetitive voltage-dependent NMDA receptor antagonist, targeting principally NMDAR pathological hyperfunction^26^, and approved in 2003 by FDA as a treatment option for moderate to severe AD.

**Figure 1:**
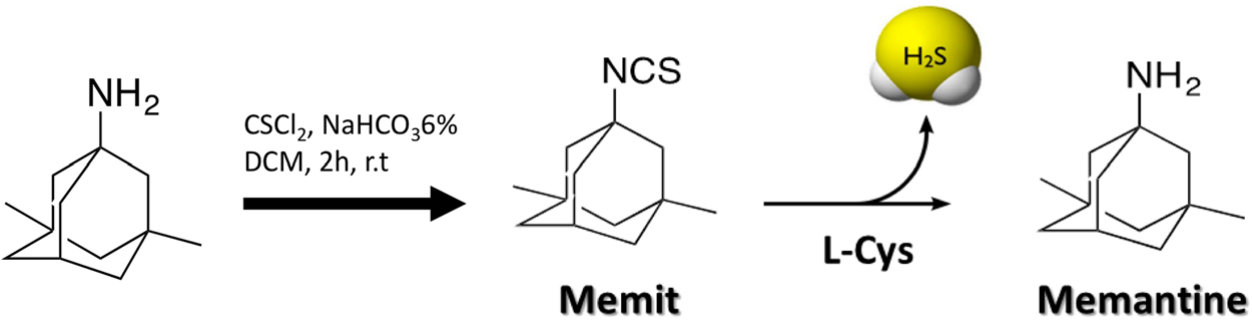
Structures of Memantine and the relative H2S-donor hybrid memit.

Starting from memantine, we replaced the free amine group with an isothiocyanate functionality as a putative H_2_S-donor moiety (Fig. 1). Indeed, recent studies showed that both synthetic and naturally occurring isothiocyanates generate H_2_S with a slow releasing rate^27,28^. Increasing studies have shown that H_2_S-generating agents for clinical use should retain some requirements such as a slow releasing rate, low and long-lasting concentration and favorable pharmacokinetics^29,30^. All these features are essential to take full advantage of the beneficial effects of H_2_S, thus avoiding the onset of toxicity.

Accordingly, a slow–releasing H_2_S donor memantine-like molecule, namely Memit, was developed and its neuroprotective properties were investigated in human neuronal-like cells.

## Methods

### Chemical synthesis

Commercial grade anhydrous solvents were used without further drying. Commercially available chemicals were purchased from Sigma Aldrich and used without further purification. Evaporation was performed in vacuum (rotating evaporator). Anhydrous Na_2_SO_4_ was used as the drying agent. Flash chromatography was performed on Merck 60 Å high-purity grade silica gel (0.40–63 μm). Reaction was followed by TLC, performed on Merck aluminum silica gel (60 F254) sheets. Spots were viewed under a UV lamp (254 nm). ^1^H and ^13^C NMR spectra were obtained using a Bruker Avance 400 spectrometer and were recorder at 400, and 101 MHz, respectively. Chemical shifts are reported in parts per million (ppm) d values, coupling constants *J* are reported in hertz (Hz). Data for ^1^H NMR spectra are reported as follows: chemical shift (δ ppm), multiplicity, integration, coupling constant (Hz). Multiplicities are reported as follows: s = singlet, m = multiplet. Synthesis of memit was reported in Fig. 1. Briefly, to a stirred solution of Memantine (300 mg, mmol 1.67) in DCM (15 ml) and NaHCO_3_ 6% (15 ml) cooled at 0 °C, was added dropwise thiophosgene (1.28 ml, 16.7 mmol). The resulting mixture was stirred at 2 h at r.t, followed by TLC, then the aqueous phase was separated and extracted several times with DCM, dried over anhydrous Na_2_SO_4_ and concentrated. The crude product was purified through flash chromatography (AcOEt/n-hexane 9:1 as the eluent) to get Memit as a clear oil^31^. 1H NMR (CDCl_3_–400 MHz): δ 0.84 (s, 6H), 1.12 (s, 2H), 1.24–1.34 (s, 4H), 1.47–1.69 (m, 4H), 1.77–1.78 (m, 2H), 2.11–2.16 (m, 1H) ppm. 13 C NMR (CDCl_3_–400 MHz): 129.96 (NCS); 59.71; 49.84; 49.59; 42.24; 42.03; 41.85; 32.78; 32.55; 28.86; 28.75; 29.64.

### Cell cultures differentiation into neurons

H9-derived Neural Stem Cells (NSCs) were cultured as reported before^32,33^. Neurobasal serum-free medium with 5 µM retinoic acid was used to differentiate cells into neurons^32,33^.

### Cellular model of inflammation and viability/proliferation assays in neuronal-like cells

The compounds Memit and Memantine were diluted in DMSO. Neuronal-like cells, differentiated from H9-derived were incubated for 16 h with an inflammatory cocktail of LPS and TNF-α (50 ng/ml), as previously reported^32^. To verify the protective effects of Memit and Memantine, neuronal-like cells were treated with the two compounds for 24 or 72 h, then the samples were washed and incubated with LPS plus TNF-α for 16 h^32^ or oligomeric Aβ_1–42_ for 24 h. In selected experiments, NaHS was used as a positive control. Following incubation, cell viability was determined using the MTS assay^32^.

### Cell proliferation/viability assays on rat microglia cells

Rat microglia cells were isolated from mixed cell culture obtained from Sprague-Dawley rat cortex, by gentle physical shaking, as described^34,35^. After isolation, cells were seeded into 96 well-plated with fresh culture media and pretreated with Memit or Memantine (10 µM) for 24 h; before washing and incubation with oligomeric Aβ_1–42_ for 24 h. Following incubation time, cell viability was determined using the MTS assay^32^.

### ROS Production

ROS activity within the cells was determined using the fluorogenic dye 2′,7′-dichlorofluorescin diacetate (H2DCFDA, Molecular Probes, Invitrogen). NSCs were seeded in black 96-multiwell plates (5 × 10^3^ cells/well). The cells were treated with Memit or Memantine at the concentration of 10 µM for 24 h; after the incubation time, cells were washed and incubated with LPS and TNF-α for 16 h. One hour prior to treatment completion, 50 μM H2DCFDA was added to the same media in the dark at 37 °C. As a positive control, H_2_O_2_ was added at a concentration of 100 μM. The fluorescence intensity (excitation 485 nm and emission 520 nm) was normalized based on the number of cells stained with crystal violet^36^. In selected experiments, NaHS was used as a positive control.

### Glioma cell culture and viability experiments

U-87MG cell line was obtained from the National Institute for Cancer Research of Genoa (Italy) and cultured as previously described^37^. The effects of compound treatment on U87MG cell viability were evaluated using the tetrazolium dye (MTT) colorimetric assay. Briefly, Memantine (10 μM) or Memit (10 μM) was added to 96-well plates containing 5,000 U-87MG cells/well. After incubation for 24 h, cell growth was measured by MTT (3-(4,5-dimethylthiazolyl-2)-2,-diphenyltetrazoliumbromide) proliferation assay. Absorbance at 570 nm was measured using a Bio-Rad model 550 microplate reader (BioRad Molecular Bioscience Group, Hercules, USA). The data represent the means of three independent experiments.

### Western blot analysis

U-87MG cells were seeded in six-well plates at a density of 1 × 10^6^/well and grown to 70–80% of confluence with standard medium (DMEM-High Glucose). Cells were treated with vehicle (0.1% DMSO) or test compounds (i.e. 10 μM Memantine or Memit, and 1 μM Rapamycin) and incubated at 37 °C for 4 and 24 h. After washing with ice-cold PBS cells were lysed and proteins (30–40 μg) were separated on Criterion TGXTM gel (4–20%) and transferred to Immuno-PVDF membrane (Bio-Rad, Milan, Italy). The membranes were probed overnight at 4 °C with primary antibodies [1:1000, LC3A/B; p62; mTOR, p-mTOR; Akt, p-Akt(Ser 473); β-actin, Cell Signaling]. The primary antibodies were detected using appropriate secondary antibody. The peroxidase was detected using a chemiluminescent substrate (ECL, Perkin Elmer). Immunoreactive bands were quantified performing a densitometric analysis with Image Lab Software (Bio-Rad, Milan, Italy) and normalized to β*-*actin.

### [^3^H]MK-801 Binding Assay

Crude synaptic membranes were prepared from the cerebral cortex of Sprague–Dawley rats^38^. Animals used procedures complied with NIH publication n° 80–23 revised in 1996 and approved by the Animals Care Commettee of the University of Pisa in compliance with the Directive 2010/63/EU on animal welfare. The pellets were stored at −80 °C for at least 24 h, and washed three more times with Tris–HEPES buffer (Tris 4.5 mM, HEPES 5 mM, pH 7.4) to remove the endogenous amino acids before the binding assay^38^.

In the binding assay, 50 μL of membrane preparation (40–50 μg protein), and 10 μL of compound were mixed at 25 °C in the presence of 50 µM L-glutamate (10 μM) and 50 μL of glycine (10 μM). Then, 50 µl of [^3^H]MK-801 (final concentration 3 nM) were added to the preparation. Tris–HEPES buffer was added to a final volume of 0.5 mL^38^. Following incubation time of 2 h at 25 °C, binding was terminated by rapid filtration. Radioactivity was measured using a PerkinElmer liquid scintillation counter. Nonspecific binding was determined in the presence of unlabeled 100 μM MK-801. The dissociation constant (Kd) of [^3^H]MK-801 in rat cortex membranes was 4.0 nM. For compound activity determination, aliquots of membrane pellets were incubated with different ligand concentrations of Memit or Memantine (10 nM-10 µM) in the absence or presence of 4 mM Cysteine for 30 min, and then incubated with 3 nM [^3^H]MK-801 for 2 h at 25 °C. Samples were then filtered, and the radioactivity was counted.

### Thioflavin T fluorescence assay

The ThT stock solution was prepared by adding 8 mg ThT to 10 mL phosphate buffer (10 mM phosphate, 150 mM NaCl, pH 7.0) and filtered through a 0.2 μm syringe filter. This stock solution should be stored in the dark and is stable for about one week. On the day of the analysis, the stock solution was diluted into PBS to obtain the concentration of 10 µM. The Aβ_1–42_ oligomers were prepared by diluting the stock solution in PBS and the dilution was shaked for 48 hours at 37 °C. The cells were seeded in black 96-multiwell plate (3000 c/w) and treated with the compounds Memit or Memantine (10 µM) for 24 h. Following incubation, the samples were washed and incubated with Aβ_1–42_ oligomers for 24 h. After this time, 200 µl of ThT 10 µM were added to each well in the dark. The ThT fluorescence intensity of each sample was recorded every 5 min using a spectrophotometer by excitation at 355 nm and emission 535 nm.

### Determination of H_2_S release by amperometric assay

The characterization of the potential H_2_S-generating properties of Memit has been carried out by an amperometric approach, through the Apollo-4000 free radical analyzer (WPI) detector and H_2_S-selective mini-electrodes. 6 different recordings have been carried out at RT. Following the manufacturer’s instructions, a “PBS buffer 10x” was prepared (NaH_2_PO_4_•H_2_O 1.28 g, Na_2_HPO_4_•12H_2_O 5.97 g, NaCl 43.88 g in 500 ml H_2_O) and stocked at 4 °C. Immediately before the experiments, the “PBS buffer 10x” was diluted using distilled water (1:10) to obtain the assay buffer and the pH adjusted to 7.4. The H_2_S-selective mini-electrode was equilibrated in 10 ml of the assay buffer, until the recovery of a stable baseline. Then, 100 µL of a DMSO solution of the tested H_2_S-releasing compounds (Memit and positive drug GYY4137) was added, at the final concentration of 1 mM; the final concentration of DMSO in the assay buffer was 1%. The generation of H_2_S was observed for 20 min. Preliminary experiments demonstrated that DMSO 1% did not produce any interference on the amperometric recording. When required by the experimental protocol, L-Cysteine (final concentration 4 mM) was added 10 min before the addition of tested compounds. L-Cysteine alone did not produce any amperometric response. The correct relationship between the amperometric currents (recorded in pA) and the corresponding concentrations of H_2_S was previously determined by suitable calibration curves, which were obtained by the use of the H_2_S donor NaHS (1–3–5–10 μM) at pH 4.0. The curves relative to the progressive increase of H_2_S vs time, following the incubation of the tested compounds, were analysed following the same procedure previously described^29^.

### Statistical analysis

The data were analysed and elaborated by Graph-Pad Prism 6.0 (GraphPad Software Inc., San Diego, CA, USA). All data are presented as the means ± SEM. Statistical analysis was performed by one-way analysis of variance (ANOVA), followed by Student–Newman–Keuls multiple comparison post hoc test or by Bonferroni’s corrected t-test for post-hoc pair-wise comparisons.

## Results

### Design and Synthesis

Memit was developed as a novel NMDAR antagonist analogue of memantine provided of H_2_S-releasing properties. This new compound was efficiently synthesized by replacing the amine functionality of memantine with an isothiocyanate group through reaction with thiophosgene in heterogeneous phase (NaHCO_3_ 6%: DCM 1:1).

### Evaluation of Memit H_2_S-releasing properties via Amperometric assay

**Figure 2:**
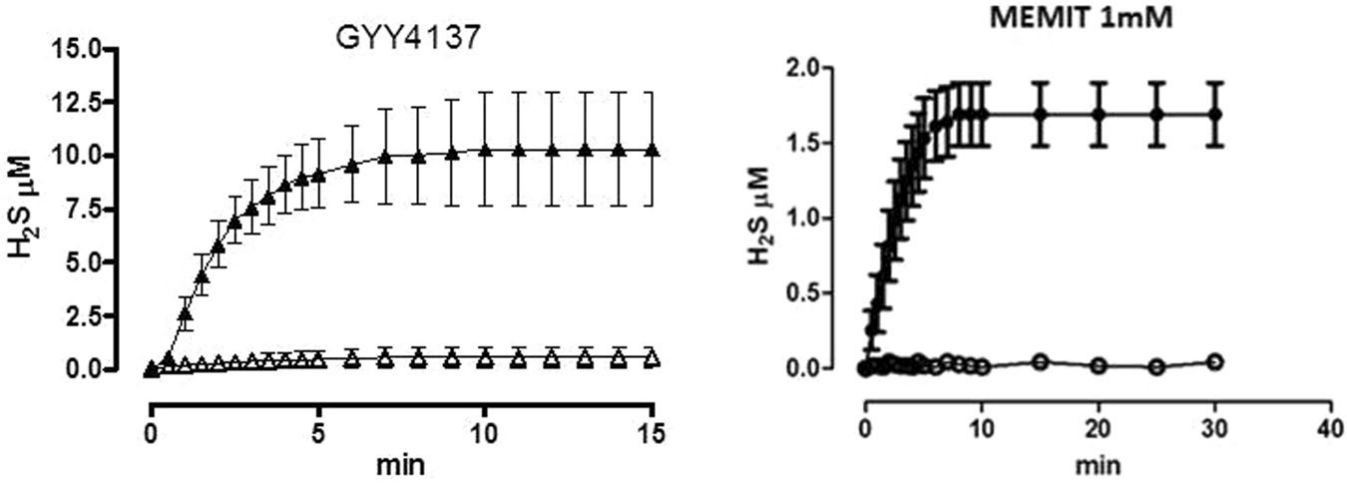
Curves describe the increase of H2S concentration, with respect to time, following the incubation of GYY4137 (1 mM) and the test compound Memit (1 mM) in the assay buffer, in the absence (white symbol) or in the presence of L-cysteine (black symbol). The vertical bars indicate the SEM.

As previously described^29^, a real-time determination of the H_2_S release was achieved by using a H_2_S-selective minielectrode incubating reference H_2_S donor (GYY4137) or Memit in the presence or in absence of L-Cysteine. As shown in Fig. 2, in the absence of L-Cys the H_2_S generation from both compounds was almost negligible, whereas after pre-incubation with L-Cys a time-dependent increasing generation of H_2_S was observed.

### Memit induces neuroprotection in human neuronal cell

**Figure 3:**
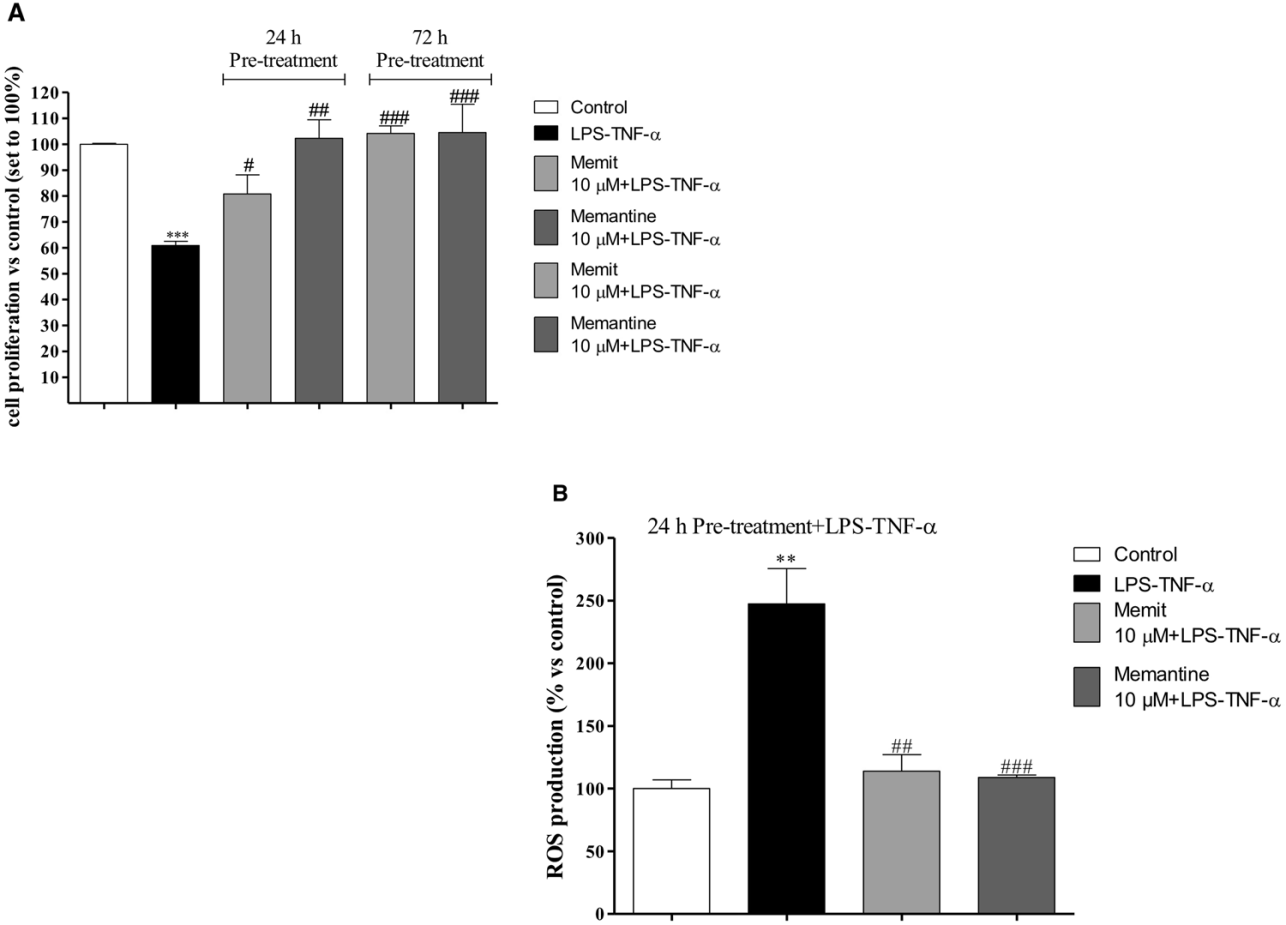
(**A**) Effect of Memit and memantine treatments on an experimental model of neuroinflammation. The cells were challenged with Memit or memantine (10 μM) for 24 h or 72 h, and then treated LPS plus TNF-α for an additional 16 h. Cell proliferation was measured by MTS assay. The data are expressed as percentages relative to untreated cells (control), which were set at 100% (mean ± SEM, N = 3). (**B**) The cells were treated as in A. After treatment, ROS levels were quantified as described in the experimental section. The data are expressed as percentages relative to untreated cells (control), which were set at 100% (mean ± SEM, N = 3). **p < 0.01, ***p < 0.001 vs control; ^#^p < 0.05, ^##^p < 0.01, ^###^p < 0.001 vs cells treated with LPS/TNF-α.

 Neuronal-like cells, obtained from H9-derived human neural stem cells (NSCs), were incubated with an inflammatory cocktail (lLPS plusTNF-α) in order to mimic *in vitro* neuroinflammation^39^. The effects of Memit (10 μM) and native drug memantine (10 μM) under physiological conditions and under inflammatory stress exposure were investigated by cellular viability experiments (Fig. 3A). The compounds did not alter cellular proliferation “per se” (data not shown). As expected, the inflammatory mixture significantly affected the proliferation of neuronal-like cells. Pre-treatment for 24 or 72 h of neuronal-like cells with the selected compounds significantly counteracted the decrease in cell proliferation elicited by the inflammatory insult. These results suggest that the new compound exerts neuro-protective effects in an experimental model of inflammation.

### Memit reduces ROS production

To investigate the scavenger properties of memit, ROS levels were measured in neuronal-like cells under inflammatory conditions. Figure 3B shows that challenging neuronal-like cells with LPS-TNF-α significantly enhanced ROS accumulation. Pre-treatment with compound memit or with the native drug for 24 h almost completely counteracted inflammation-mediated ROS accumulation, thus suggesting that both compounds are able to prevent ROS accumulation.

### Memit inhibits Aβ(1-40) self-induced aggregation

**Figure 4:**
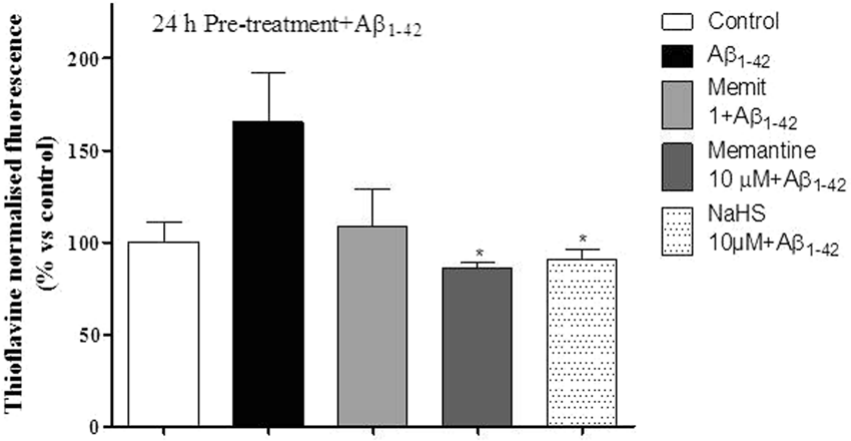
Effect of the anti-amyloidogenic compounds, Memit and memantine, on amyloid oligomers formation. The cells were pre-treated with Memit, Memantine or NaHS (10 µM) for 24 h. Following incubation time, cells were washed and incubated with Aβ1-42 oligomers for 24 h. Following incubation, 200 µl of ThT 10 µM were added to each well in the dark. The ThT fluorescence intensity of each sample was recorded every 5 min using a spectrophotometer by excitation at 355 nm and emission 535 nm. Values represent mean ± SEM; *p < 0.05 vs Aβ1-42.

Accumulation of extracellular deposits of β-amyloid and abundant neurofibrillary tangles in the brain are correlated with neuron loss and progressive dementia^15^. Therefore, in order to evaluate the influence of memit on amyloid fibrils, a Th-T fluorescence assay was performed using NaHS as positive control^15,16,40^. Memit and Memantine were tested at 10 µM concentration; the results are reported in Fig. 4. As expected, Aβ1-42 significantly enhanced ThT-related fluorescence (165.5 ± 26.5% versus control). All the tested compounds exerted a significant inhibition of ThT-related fluorescence, thus indicating inhibition of Aβ1-42 self-aggregation. In particular Memit induced an Aβ1-42 self-aggregation inhibition comparable to its progenitor Memantine.

### Memit induces autophagy in U-87MG cell line

**Figure 5:**
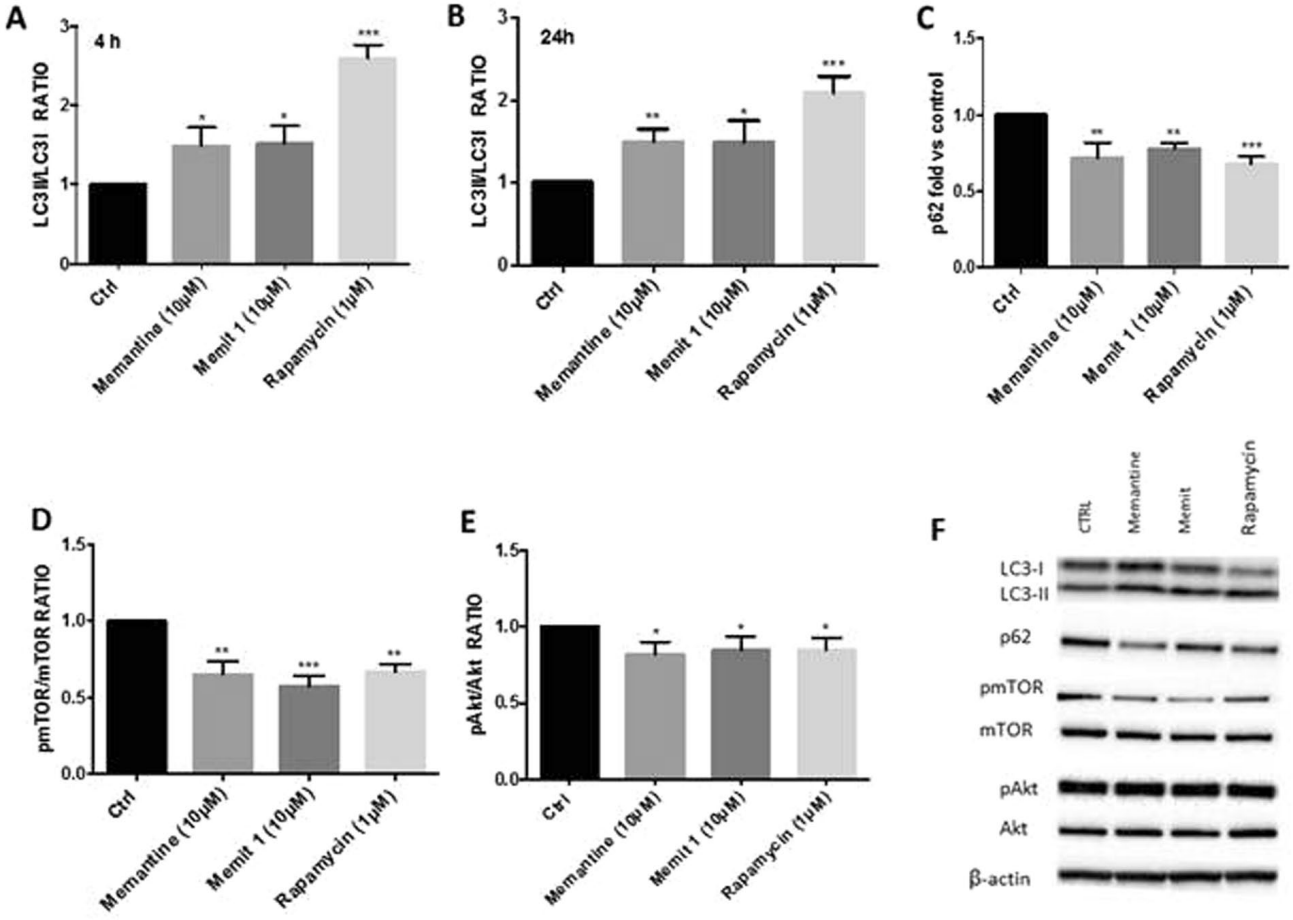
Western blot quantification of LC3II/LC3I ratio, p62, mTOR and Akt as hallmarks of the degree of ATG activation in U87MG cells. Memantine (10 mM), Memit (10 mM) and Rapamycin (1 mM) induced increased LC3II/LC3I ratio at 4h (**A**) and 24h (**B**); a significant p62 degradation (**C**) and decreased p-mTOR/mTOR ratio (**D**) and pAkt/Akt ratio (**E**). Results represent mean ± SEM of three different gels. *p < 0.05, **p < 0.01, ***p < 0.001 versus vehicle treated cells (Control). Representative western blots are shown in Panel F.

**Figure 6:**
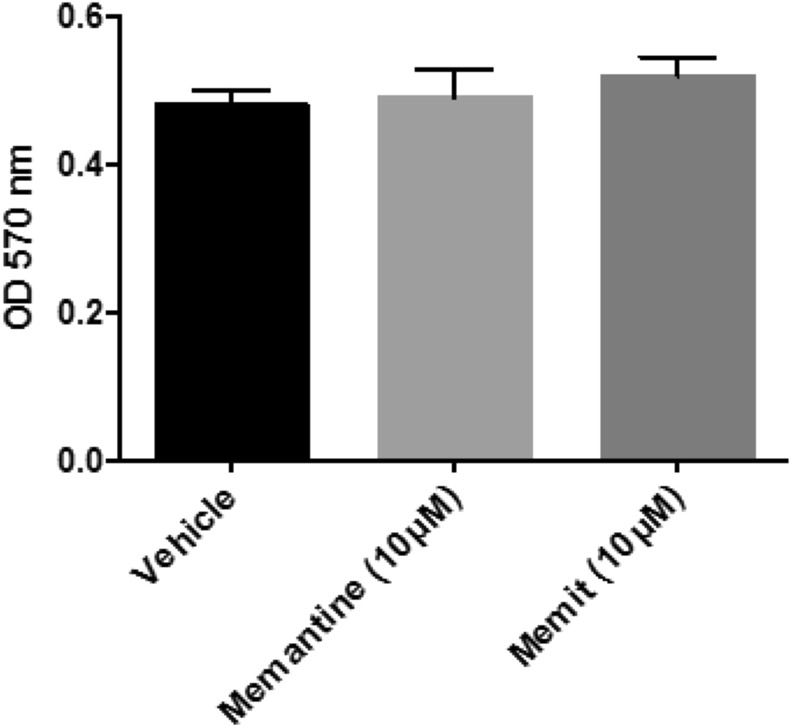
Cell viability assay. MTT assay showing U-87MG cells viability after 24 h treatment with tested compounds. Results are expressed as the mean ± SEM of twelve biological replicates.

Autophagy (ATG) is a complex and finely regulated pathway, essential for the correct cellular physiology as it is involved in the degradation of long-lived cytoplasmic proteins, protein complexes, or damaged organelles. Impairments of the autophagic process are associated with several neurodegenerative disorders^41^, it is thus possible that the induction of ATG may be exploited as a strategy to assist neuronal survival. The mammalian target of Rapamycin (m-TOR) is an evolutionarily conserved serine-threonine kinase that is known to play a major role in the modulation of ATG^42,43^. Therefore, to explore the neuroprotective potential of Memit and Memantine we decided to evaluate their ability to induce ATG in U-87MG cells, a human glioblastoma cell line characterized by a weak ATG machinery due to the upregulation of m-TOR^44–46^. Expression of protein indicators of autophagy, such as LC3-II^47^, p62^48^ and m-TOR, was detected by western blotting, using Rapamycin as a positive control. As shown in Fig. 5 a significant up-regulation of LC3II expression was observed after 4 h treatment with 10 μM Memit or Memantine (Fig. 5A), and also remained almost unchanged until 24 h treatment (Fig. 5B), suggesting that both Memit and Memantine are able to stimulate the ATG flux. Consistently, after 4 h treatment parallel decreased expressions of p62, which is degraded during autophagy, and p-mTOR were observed (Fig. 5C,D). Next, we decided to address whether the observed induction of autophagy in U-87MG cells caused by treatment with 10 μM Memit or Memantine was accomplished via inhibition of the survival PI3K/Akt/mTOR pathway^49^. To this end, we analyzed the phosphorylation status of Akt in U-87MG cells treated with test compounds. As shown in Fig. 5E, 4 h treatment with Memit and Memantine (10 μM) resulted also in a significant reduction of the phosphorylation levels of Akt-Ser 473 (*P < 0.05, **P < 0.01, versus vehicle treated cells), suggesting that, similarly to Rapamycin, both compounds may induce autophagy through the inhibition of the mTOR phosphorylation by the PI3K/AKT/mTOR pathway (full length Western blots for Fig. 5F are shown in Supplementary Fig. S1). In addition, cellular viability was determined using the MTT colorimetric assay. No significant alterations of cell viability were observed in U-87MG cells treated for 24 h with (10 μM) test compounds, as compared to vehicle (0.1% DMSO) (Fig. 6).

### Evaluation of Memit binding activity on NMDAR

**Figure 7:**
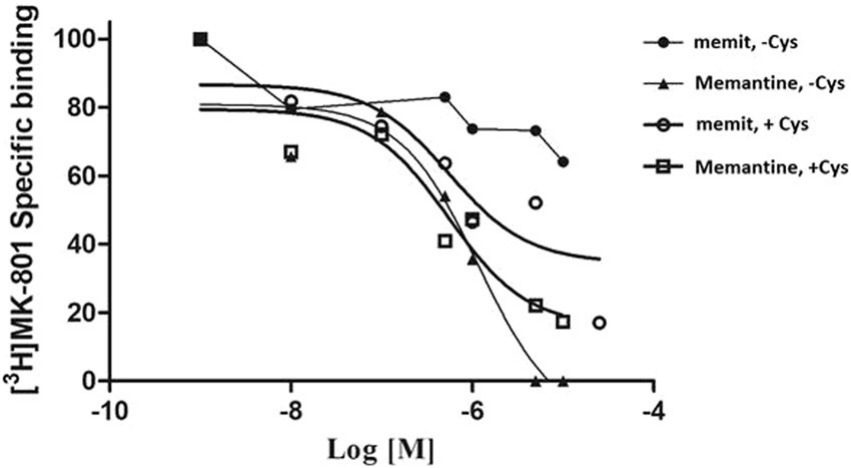
Displacement of specific [3H]MK-801 binding in rat cortex membranes in the absence or presence of cysteine (Cys). Rat cortex membranes were incubated with the indicated compounds and [3H]MK-801, in the absence or presence of Cys, Data are reported as percentage of radioligand specific binding and are the means ± S.E.M. of three different experiments (performed in duplicate).

Memit was tested to assess its ability to inhibit native-target activity (i.e. NMDAR). Binding to NMDAR was assessed using a radioligand assay on cortex membranes based on the displacement of [3H]MK-801^38^, in the presence or absence of Cys (Table 1). Interestingly, while Memantine showed a significant displacement of [3H]MK-801 both in presence or in absence of Cys (Ki = 954.8 ± 74.2 nM and 328.8 ± 10.9 nM respectively), our H_2_S donor compound revealed a significant activity only in the presence of an organic source of H_2_S (Fig. 7 and Table 1). Importantly, the Ki value for memit (458.4 ± 77.7 nM) was comparable to that obtained for Memantine. These data demonstrate that memit could be considered a pro-drug of Memantine, released in the presence of Cys, and that the chemical modification did not alter the compound ability to bind NMDA.

**Table 1:** Displacement of specific [^3^H]MK-801 binding in rat cortex membranes in the absence or presence of cysteine (Cys).

Compound	Ki, nM (−Cys)^a^	Ki, nM (+Cys 4 mM, 30 min)^a^
Memit	28.0%^b^	458.4 ± 77.7
Memantine	954.8 ± 74.2	328.8 ± 10.9

Data are reported as the means ± S.E.M. of three different experiments (performed in duplicate). ^a^The Ki values are means ± SEM derived from an iterative curve-fitting procedure (Prism program, GraphPad, San Diego, CA). ^b^Percentage of inhibition is reported for Memit in the absence of Cys.

Of note, Memit itself increased significantly its affinity for NMDA receptor in the presence of Cys (Fig. 7, Table 1), suggesting that Cys is involved in the regulation of the receptor affinity/functionality. Consistent with our hypothesis, crucial cysteine residues have been suggested to be involved in the modulation of NMDA protein activity and signaling events via other reactions of thiol groups^50^. In particular, the presence of cysteine in the binding buffer could involve reactions that affect ligand binding allosterically, as can be also observed by the Ki values of reference drug in the presence and in absence of Cys^50^.

### Memit protects neuronal-like cells and microglia from Aβ(1-42) induced injury

**Figure 8:**
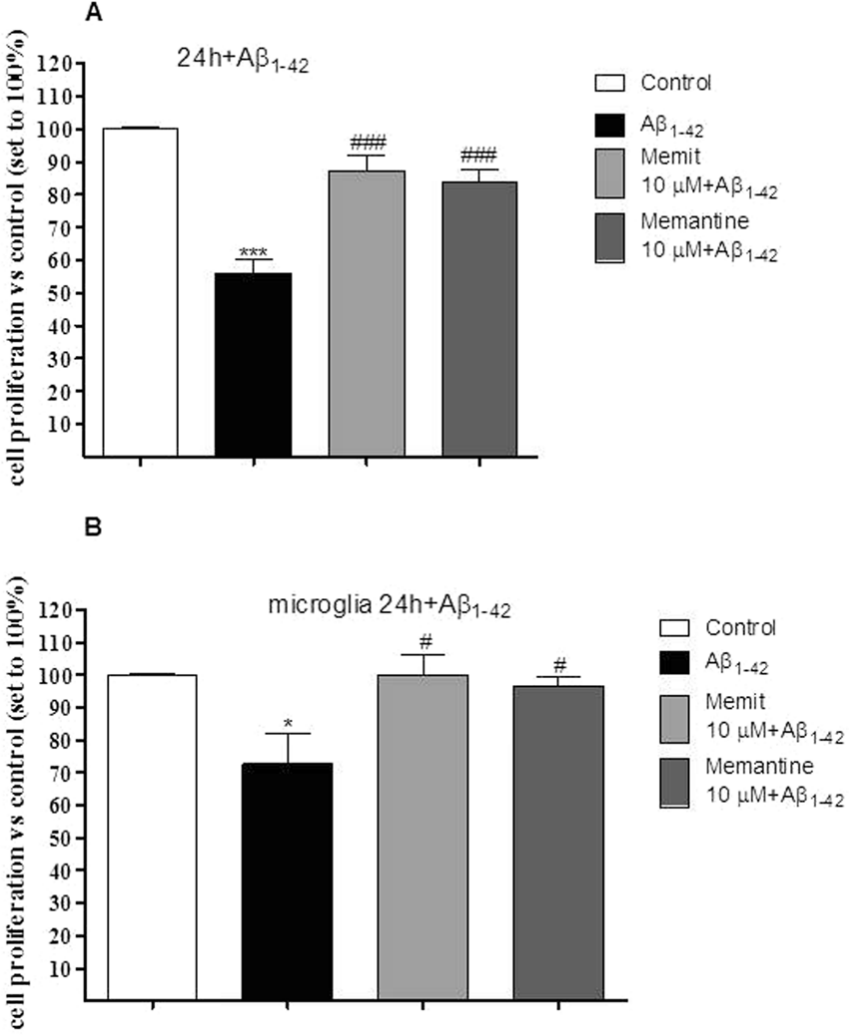
Neuronal-like cells (panel A) or rat microglia cells (panel B) were pre-treated with Memit or Memantine (10 μM) for 24 h. After washing, the cells were challenged with Aβ1-42 for 24 h. At the end of treatments, cell proliferation was measured by MTS assay. The data are expressed as percentages relative to untreated cells (control), which were set at 100% (mean ± SEM, N=3) *p<0.05 vs control; ^#^p<0.05 vs cells treated with Aβ1-42.

Several studies have proved that H_2_S and NMDA ligands have protective effects on Aβ-induced cellular injury^15,16^. On this basis, the effect of Memit on injured neurons was assessed. As expected, oligomeric Aβ caused a significant reduction of neuronal viability (Fig. 8, panel A). When cells were pre-challenged with Memit, a marked cyto-protective effect was observed (Fig. 8, panel A). Similar results were obtained probing cells with Memantine (Fig. 8, panel A).

Microglia, together with astrocytes, forms the main active immune defense of the CNS. During the inflammation process, induction of NF-κB occurs, accompanied by a release of inflammatory mediators such as TNF-α, IL-6 and nitrite ions. Notably, also levels of CBS and H_2_S result down-regulated^51,52^. Consistently, to further determine the neuroprotection elicited by our hybrid, we decided to evaluate effects of Memit and Memantine on rat microglia cells. Cells were pretreated with the two compounds (10 µM concentration) followed by incubating Aβ_1–42_ oligomers. As depicted in Fig. 8 (panel B), pre-treatment with Memit and Memantine completely restored cell proliferation. These data demonstrate that the compounds can protect both neuronal cells and microglia from Aβ-induced injury.

## Discussion

The goal of the present work was to develop a new and improved molecule for the treatment of neurodegeneration by modifying the structure of a well-established drug that is already approved for therapeutic use. Thus, starting from memantine, a compound currently prescribed for the treatment of moderate-to-severe Alzheimer’s disease, we created a new chemical entity, named memit, by introducing an isothiocyanate moiety capable of releasing H_2_S into its scaffold.

Although the pharmacological profile of memantine has been extensively studied (moderate affinity, uncompetitive, voltage-dependent, NMDA receptor antagonist that exhibits fast on/off kinetics^53^), the conjunction with an H_2_S-donor moiety creates a new hybrid molecule whose properties need to be fully investigated. Putatively such a molecule should exert neuroprotective and anti-inflammatory effects through both hydrogen sulphide- and NMDA-mediated mechanisms. This could result in a delaying of the neurodegeneration process.

Therefore, our initial studies were directed to assess whether memit still retains the pharmacological profile of the “native drug”, while also being a source for H_2_S in the CNS. To evaluate its H_2_S donor properties we used an amperometric approach^29^, which provides a real-time determination of H_2_S-release, either in the absence or in the presence of an organic thiol, such as L-cysteine. The results of the amperometric analysis on memit showed a prolonged and persisting L-cysteine mediated release, similar to that exhibited by the reference slow H_2_S-releasing agent GYY4137, a phosphinodithioate derivative.

Next, we investigated the neuroprotective activity of memit in human neuronal-like cells and in rat microglia cells, with the former representing the main active immune defense of the CNS. In neuronal cells, the neuroprotection was evaluated following an LPS-TNFα insult, known to induce oxidative stress and consequently apoptosis and cell death. The new drug demonstrated to protect cells from both oxidative stress and cytotoxicity induced by LPS-TNFα. The observed effects can be attributed to both NMDA binding and H_2_S release. Indeed, hydrogen sulfide has been proven to attenuate neural injury and cytokine release in rats^54^ and memantine has been shown to mitigate colchicine-induced inflammation in the hippocampus^55^.

In addition, Memit was able to reduce the Aβ(1-42) self-induced aggregation, with effects comparable to those of its progenitor memantine. Accordingly, memit and the native drug were also able to reduce Aβ oligomers-induced damage in rat microglia cells. Moreover, the observed reduction in Aβ accumulation elicited by Memit was shown to protect neuronal cells from the peptide-induced toxicity.

Consistent with our data, an over activation of NMDA receptor has been linked to abnormal Aβ deposition in AD and memantine has been demonstrated to decrease Aβ secretion in human cells or transgenic mice^56,57^.

Interestingly, H_2_S has been also proven to inhibit protein fibrillation of Aβ peptides by forming trisulfide bridges^58^ and therefore it may contribute to Memit-elicited properties.

The observed cytoprotection from Aβ-induced toxicity was then tested in microglia cells. Recent evidences have highlighted the pivotal role of these cells in different neuropathologies, including AD^59^. Notably, pharmacological studies suggest that promoting microglia polarization plays a key role in maintaining tissue homeostasis through different mechanisms including the suppression of inflammation^59^. In our hand, both memit and the native drug were also able to reduce Aβ oligomers-induced damage in rat microglia cells, suggesting the two drugs as promising protective agents for non-neuronal cells too.

Moreover, since a large body of evidence underlies the existence of a correlation between neurodegeneration and altered autophagic flux^60^, we also decided to investigate the ability of memit and memantine to induce autophagy in U87-MG cells. Our western blot analysis revealed that both compounds were able to significantly affect the expression of common autophagy markers such as LC3II, p62, mTOR and Akt, suggesting that autophagy could be an additional mechanism to strengthen their efficacy as neuroprotective drugs. These data are of particular relevance considering the role of AKT/mTOR pathway in the modulation of autophagy and the clearance of protein oligomers in neurodegenerative diseases^49^.

Finally, we also tested the ability of memit to bind to the NMDA receptor, the main target of memantine. Interestingly, we found that memit displayed an affinity comparable to that of the native drug only when cysteine was present in the assay medium, while no binding was observed in the absence of cysteine. These observations suggest that memit behaves as a prodrug, and that the binding is due to the production of memantine. In this sense, it has to be mentioned the reported facilitating effects of hydrogen-sulfide on NMDA receptors^61,62^. Nevertheless, the functional consequences of H_2_S-mediated NMDA receptor activation in neurons and stem cells is not strictly linked to neurotoxicity. For example, these facilitating actions are age- and receptor subunit-specific^61^ and can finally promote synaptic plasticity. Furthermore, H_2_S has been demonstrated to enhance NMDA receptor-mediated currents and facilitate the induction of hippocampal long-term potentiation (LTP)^63^. Finally, activation of NMDA receptors increases proliferation and differentiation of hippocampal neural progenitor cells^64^ and thus does not show toxic effects on differentiated cells.

Further studies will be needed to elucidate changes in NMDA receptor functionality that can be mediated by Memit-released hydrogen sulfide.

## Conclusion

Unfortunately, to date the etiopathology of neurodegeneration is still not well understood and the therapies that are currently available lack in efficacy, as they are unable to completely block neuronal damage. Consequently, there is an enormous need for new therapeutic tools that are at least capable of slowing down the progression of the pathology. The work we presented here describes the synthesis and the preliminary *in vitro* evaluation of an innovative multi-functional prodrug. To the best of our knowledge, memit is the first compound capable of producing H_2_S in the CNS that was designed starting from the well-known drug memantine. The new compound we have developed has the potential of restoring H_2_S levels in the CNS and, like memantine, has the ability of inducing autophagy, thus eliciting neuroprotective effects. However, further *in vivo* investigations will be necessary to fully appreciate the synergistic or cumulative effects due to the H_2_S-releasing moiety and the native drug.

## Supplementary information

Supplementary figure

## References

[1] Morrison LD, Smith DD, Kish SJ (1996). Brain S‐adenosylmethionine levels are severely decreased in Alzheimer’s disease. Journal of neurochemistry.

[2] Zhang X, Bian J-S (2014). Hydrogen sulfide: A neuromodulator and neuroprotectant in the central nervous system. ACS chemical neuroscience.

[3] Abe K, Kimura H (1996). The possible role of hydrogen sulfide as an endogenous neuromodulator. Journal of Neuroscience.

[4] Liu X, Jiang P, Huang H, Yan Y (2008). Plasma levels of endogenous hydrogen sulfide and homocysteine in patients with Alzheimer’s disease and vascular dementia and the significance thereof. Zhonghua yi xue za zhi.

[5] Kimura H (2002). Hydrogen sulfide as a neuromodulator. Molecular neurobiology.

[6] Kimura H (2013). Physiological role of hydrogen sulfide and polysulfide in the central nervous system. Neurochemistry international.

[7] Nagai Y, Tsugane M, Oka J-I, Kimura H (2004). Hydrogen sulfide induces calcium waves in astrocytes. The FASEB journal.

[8] Giuliani D (2013). Hydrogen sulfide slows down progression of experimental Alzheimer’s disease by targeting multiple pathophysiological mechanisms. Neurobiology of Learning and Memory.

[9] Sestito S, Nesi G, Pi R, Macchia M, Rapposelli S (2017). Hydrogen sulfide: a worthwhile tool in the design of new multitarget drugs. Frontiers in Chemistry.

[10] Qu K, Lee S, Bian J, Low C-M, Wong P-H (2008). Hydrogen sulfide: neurochemistry and neurobiology. Neurochemistry international.

[11] Wei H-J, Li X, Tang X-Q (2014). Therapeutic benefits of H2S in Alzheimer’s disease. Journal of Clinical Neuroscience.

[12] Kimura Y, Goto Y-I, Kimura H (2010). Hydrogen sulfide increases glutathione production and suppresses oxidative stress in mitochondria. Antioxidants & redox signaling.

[13] Jia J (2013). Differential mechanisms underlying neuroprotection of hydrogen sulfide donors against oxidative stress. Neurochemistry international.

[14] Xie Z-Z (2014). Sulfhydration of p66Shc at cysteine59 mediates the antioxidant effect of hydrogen sulfide. Antioxidants & redox signaling.

[15] Liu Y, Bian J (2010). Hydrogen sulfide protects amyloid-β induced cell toxicity in microglia. Journal of Alzheimer’s disease: JAD.

[16] Fan H (2013). Hydrogen sulfide protects against amyloid beta-peptide induced neuronal injury via attenuating inflammatory responses in a rat model. Journal of Biomedical Research.

[17] Hu LF, Wong PTH, Moore PK, Bian JS (2007). Hydrogen sulfide attenuates lipopolysaccharide‐induced inflammation by inhibition of p38 mitogen‐activated protein kinase in microglia. Journal of neurochemistry.

[18] Moore PK, Bhatia M, Moochhala S (2003). Hydrogen sulfide: from the smell of the past to the mediator of the future?. Trends in pharmacological sciences.

[19] Chen Z, Han L, Xu M, Xu Y, Qian X (2013). Rationally designed multitarget anticancer agents. Current medicinal chemistry.

[20] Trstenjak U, Kikelj D (2011). Multitarget cardiovascular drugs. Curr Med Chem.

[21] Bolognesi ML, Matera R, Minarini A, Rosini M, Melchiorre C (2009). Alzheimer’s disease: new approaches to drug discover. y. Current Opinion in Chemical Biology.

[22] Nesi, G., Sestito, S., Digiacomo, M. & Rapposelli, S. Oxidative Stress, Mitochondrial Abnormalities and Proteins Deposition: Multitarget Approaches in Alzheimer’s Disease. *Current topics in medicinal chemistry*, 10.2174/1568026617666170607114232 (2017). 10.2174/1568026617666170607114232 28595557

[23] Mostafa DK, El Azhary NM, Nasra RA (2016). The hydrogen sulfide releasing compounds ATB-346 and diallyl trisulfide attenuate streptozotocin-induced cognitive impairment, neuroinflammation, and oxidative stress in rats: involvement of asymmetric dimethylarginine. Canadian journal of physiology and pharmacology.

[24] Egea J (2015). Melatonin–sulforaphane hybrid ITH12674 induces neuroprotection in oxidative stress conditions by a ‘drug–prodrug’mechanism of action. British journal of pharmacology.

[25] Keri RS (2016). New Tacrine Hybrids with Natural‐Based Cysteine Derivatives as Multitargeted Drugs for Potential Treatment of Alzheimer’s Diseas. e. Chemical biology & drug design.

[26] Alam S, Lingenfelter KS, Bender AM, Lindsley CW (2017). Classics in Chemical. Neuroscience: Memantine..

[27] Citi V (2014). Hydrogen sulfide releasing capacity of natural isothiocyanates: is it a reliable explanation for the multiple biological effects of Brassicaceae?. Planta medica.

[28] Martelli A (2014). Pharmacological characterization of the vascular effects of aryl isothiocyanates: is hydrogen sulfide the real player?. Vascular pharmacology.

[29] Martelli A (2013). Arylthioamides as H2S donors: L-cysteine-activated releasing properties and vascular effects *in vitro* and *in vivo*. ACS medicinal chemistry letters.

[30] Barresi E (2017). Iminothioethers as hydrogen sulfide donors: from the gasotransmitter release to the vascular effects. Journal of Medicinal Chemistry.

[31] Klimochkin YN (1991). Synthesis and antiviral activity of sulfur-containing derivatives of adamantane. Pharmaceutical Chemistry Journal.

[32] Daniele S, Zappelli E, Martini C (2015). Trazodone regulates neurotrophic/growth factors, mitogen-activated protein kinases and lactate release in human primary astrocytes. Journal of neuroinflammation.

[33] Liu L, Liu C, Zhong Y, Apostolou A, Fang S (2012). ER stress response during the differentiation of H9 cells induced by retinoic acid. Biochemical and biophysical research communications.

[34] Barger SW, Basile AS (2001). Activation of microglia by secreted amyloid precursor protein evokes release of glutamate by cystine exchange and attenuates synaptic function. Journal of neurochemistry.

[35] Ni, M. & Aschner, M. Neonatal rat primary microglia: isolation, culturing, and selected applications. *Current protocols in toxicology*, 12.17. 11-12.17. 16 (2010). 10.1002/0471140856.tx1217s43 PMC2959194 20960423

[36] Daniele S, Da Pozzo E, Iofrida C, Martini C (2016). Human Neural Stem Cell Aging Is Counteracted by α-Glycerylphosphorylethanolamine. ACS chemical neuroscience.

[37] Bellusci L (2017). New Insights into the Potential Roles of 3-Iodothyronamine (T1AM) and Newly Developed Thyronamine-like TAAR1 Agonists in Neuroprotection. Frontiers in Pharmacology.

[38] Simoni E (2012). Combining Galantamine and Memantine in Multitargeted, New Chemical Entities Potentially Useful in Alzheimer’s Disease. Journal of Medicinal Chemistry.

[39] Daniele S, Da Pozzo E, Zappelli E, Martini C (2015). Trazodone treatment protects neuronal-like cells from inflammatory insult by inhibiting NF-κB, p38 and JNK. Cellular signalling.

[40] Zhao F-l (2016). Hydrogen Sulfide Selectively Inhibits γ-Secretase Activity and Decreases Mitochondrial Aβ Production in Neurons from APP/PS1 Transgenic Mice. Neurochemical research.

[41] Nixon RA (2013). The role of autophagy in neurodegenerative disease. Nat Med.

[42] Crino PB (2016). The mTOR signalling cascade: paving new roads to cure neurological disease. *Nature Reviews*. Neurology.

[43] Garza-Lombo C, Gonsebatt ME (2016). Mammalian Target of Rapamycin: Its Role in Early Neural Development and in Adult and Aged Brain Function. Front Cell Neurosci.

[44] Fan QW, Weiss WA (2012). Inhibition of PI3K-Akt-mTOR signaling in glioblastoma by mTORC1/2 inhibitors. Methods in molecular biology (Clifton, N.J.).

[45] Arcella A (2013). Rapamycin inhibits the growth of glioblastoma. Brain research.

[46] Catalano M (2015). Autophagy induction impairs migration and invasion by reversing EMT in glioblastoma cells. Mol Oncol.

[47] Mizushima N, Yoshimori T, Levine B (2010). Methods in mammalian autophagy research. Cell.

[48] Bjørkøy G (2005). p62/SQSTM1 forms protein aggregates degraded by autophagy and has a protective effect on huntingtin-induced cell death. The Journal of cell biology.

[49] Heras-Sandoval D, Perez-Rojas JM, Hernandez-Damian J, Pedraza-Chaverri J (2014). The role of PI3K/AKT/mTOR pathway in the modulation of autophagy and the clearance of protein aggregates in neurodegeneration. Cell Signal.

[50] Lipton SA (2002). Cysteine regulation of protein function–as exemplified by NMDA-receptor modulation. Trends in neurosciences.

[51] Wojtera M, Sikorska B, Sobow T (2005). Microglial cells in neurodegenerative disorders. Folia neuropathologica.

[52] Lee SW (2006). Hydrogen sulphide regulates calcium homeostasis in microglial cells. Glia.

[53] Alam, S., Lingenfelter, K. S., Bender, A. M. & Lindsley, C. W. Classics in Chemical Neuroscience: Memantine. ACS Chemical Neuroscience (2017). 10.1021/acschemneuro.7b00270 28737885

[54] Xuan A (2012). Hydrogen sulfide attenuates spatial memory impairment and hippocampal neuroinflammation in beta-amyloid rat model of Alzheimer’s diseas. e. J Neuroinflammation.

[55] Sil S, Ghosh T, Ghosh R (2016). NMDA receptor is involved in neuroinflammation in intracerebroventricular colchicine-injected rats. Journal of immunotoxicology.

[56] Ray B, Banerjee PK, Greig NH, Lahiri DK (2010). Memantine treatment decreases levels of secreted Alzheimer’s amyloid precursor protein (APP) and amyloid beta (A beta) peptide in the human neuroblastoma cells. Neurosci Lett.

[57] Alley GM (2010). Memantine lowers amyloid-beta peptide levels in neuronal cultures and in APP/PS1 transgenic mice. J Neurosci Res.

[58] Rosario-Alomar MF (2015). Hydrogen sulfide inhibits amyloid formation. The journal of physical chemistry. B.

[59] Gupta N (2018). Recent progress in therapeutic strategies for microglia-mediated neuroinflammation in neuropathologies. Expert opinion on therapeutic targets.

[60] Fujikake N, Shin M, Shimizu S (2018). Association Between Autophagy and Neurodegenerative Diseases. Frontiers in neuroscience.

[61] Yakovlev AV, Kurmasheva ED, Ishchenko Y, Giniatullin R, Sitdikova GF (2017). Age-Dependent, Subunit Specific Action of Hydrogen Sulfide on GluN1/2A and GluN1/2B NMDA Receptors. Frontiers in cellular neuroscience.

[62] Chen MJ (2011). Gene profiling reveals hydrogen sulphide recruits death signaling via the N-methyl-D-aspartate receptor identifying commonalities with excitotoxicity. J Cell Physiol.

[63] Kimura H (2000). Hydrogen sulfide induces cyclic AMP and modulates the NMDA receptor. Biochem Biophys Res Commun.

[64] Joo JY (2007). Activation of NMDA receptors increases proliferation and differentiation of hippocampal neural progenitor cells. J Cell Sci.

