# Supplementary material for: Memantine prodrug as a new agent for Alzheimer’s Disease
Source: Sci Rep. 2019 Mar 15;9:4612. doi: 10.1038/s41598-019-40925-8 (PMC6420495; doi:10.1038/s41598-019-40925-8)
Supplement: Supplementary file 1 — Supplementary figure [file 41598_2019_40925_MOESM1_ESM.doc]

**Memantine prodrug as a new agent for Alzheimer’s Disease**

**Simona Sestito^1^**^+^**, Simona Daniele^1^**^+^**, Deborah Pietrobono^1^, Valentina Citi^1^, Lorenza Bellusci^2^, Grazia Chiellini^2^, Vincenzo Calderone^1,3^, Claudia Martini^1^, Simona Rapposelli^1,3^**^*^

^1^Department of Pharmacy, University of Pisa, Pisa, 56126, Italy

^2^Department of Pathology, University of Pisa, Pisa, 56126, Italy

^3^ Interdepartmental Research Centre for Biology and Pathology of Aging, University of Pisa.

Corresponding Author:[simona.rapposelli@unipi.it](mailto:simona.rapposelli@unipi.it)

^+^these authors contributed equally to this work

**Supplementary Figures**

**Suppl. Figure 1**

**Supplementary Figure S1. Full-length blots relative to the cropped images showed in Figure 5.** Uncropped Western blots relative to figure 5 panel E in the manuscript are shown as indicated. The membranes themselves were cut into sections to enable probing of the same blots with multiple antibodies. Therefore the above membrane sections are the full blots for each antibody.
